# Hydrophobic cell surface display system of PETase as a sustainable biocatalyst for PET degradation

**DOI:** 10.3389/fmicb.2022.1005480

**Published:** 2022-09-29

**Authors:** Yunpu Jia, Nadia A. Samak, Xuemi Hao, Zheng Chen, Qifeng Wen, Jianmin Xing

**Affiliations:** ^1^CAS Key Laboratory of Green Process and Engineering, State Key Laboratory of Biochemical Engineering, Institute of Process Engineering, Chinese Academy of Sciences, Beijing, China; ^2^College of Chemical Engineering, University of Chinese Academy of Sciences, Beijing, China; ^3^Environmental Microbiology and Biotechnology, Aquatic Microbiology, University of Duisburg-Essen, Essen, Germany; ^4^Chemistry and Chemical Engineering Guangdong Laboratory, Shantou, China

**Keywords:** PET biodegradation, whole-cell catalysis, extracellular protein production, polyethylene terephthalate, surface display, PETase

## Abstract

Remarkably, a hydrolase from *Ideonella sakaiensis* 201-F6, termed PETase, exhibits great potential in polyethylene terephthalate (PET) waste management due to it can efficiently degrade PET under moderate conditions. However, its low yield and poor accessibility to bulky substrates hamper its further industrial application. Herein a multigene fusion strategy is introduced for constructing a hydrophobic cell surface display (HCSD) system in *Escherichia coli* as a robust, recyclable, and sustainable whole-cell catalyst. The truncated outer membrane hybrid protein FadL exposed the PETase and hydrophobic protein HFBII on the surface of *E. coli* with efficient PET accessibility and degradation performance. *E. coli* containing the HCSD system changed the surface tension of the bacterial solution, resulting in a smaller contact angle (83.9 ± 2° vs. 58.5 ± 1°) of the system on the PET surface, thus giving a better opportunity for PETase to interact with PET. Furthermore, pretreatment of PET with HCSD showed rougher surfaces with greater hydrophilicity (water contact angle of 68.4 ± 1° vs. 106.1 ± 2°) than the non-pretreated ones. Moreover, the HCSD system showed excellent sustainable degradation performance for PET bottles with a higher degradation rate than free PETase. The HCSD degradation system also had excellent stability, maintaining 73% of its initial activity after 7 days of incubation at 40°C and retaining 70% activity after seven cycles. This study indicates that the HCSD system could be used as a novel catalyst for efficiently accelerating PET biodegradation.

## Introduction

PET is a plastic material excessively used in beverage and food packaging and synthetic textile fibers (Geyer et al., 2017). The growing demand for PET requires a practical and comprehensive global manufacturing and waste management system to reduce the littering of natural systems and the usage of fossil resources. Currently, PET is mainly recycled by mechanical recycling methods after intensive sorting. Subsequently, sufficient quality is assumed, and the products can be reused in the established production processes. However, during melting and reprocessing, the chain length of the polymer is affected by shear forces and high temperatures, which can easily lead to thermal and mechanical degradation, thus changing the properties of the polymer and producing low-quality plastic (Kröll et al., 2021). A recent alternative to realize higher circularity is the chemical recycling of commodity polymers to monomers, which requires hazardous chemicals and conditions (Coates and Getzler, 2020). Although industrial PET recovery processes have been established to reuse collected material and replace virgin feedstocks, a significant proportion of the global PET waste is incinerated, buried, or entered the environment due to improper treatment.

Furthermore, plastic waste, such as PET, may be considered a potential source of carbon for industrial biotechnology because of its carbon-rich polymer characteristics. In 2016, Yoshida (Yoshida et al., 2016) tested different bacteria isolated from a bottle recycling plant’s environmental samples (e.g., wastewater, sediment, activated sludge). It has been found that one of the isolates, *Ideonella sakaiensis* 201-F6 strain, can use the plastic polymer PET as a sole carbon source to produce PETase and MHETase enzymes at low temperatures. PETase showed higher specificity and degradation performance on commercial bottle-derived PET than other previously described hydrolases, like FsC from fungus *Fusarium solani* (Martinez et al., 1992), bacterial cutinases TfH from thermophilic actinomycete *Thermobifida fusca* (Müller et al., 2005), LCC derived from leaf-branch compost metagenome (Sulaiman et al., 2012)) and so on. These two features make PETase a prospective candidate for the decomposition and recovery of PET at environmental temperature. However, there is an urgent demand to enhance further the performance of PETase in the decomposition of highly crystalline PET, which is a vital factor hindering its realization for industrial applications.

In industrial applications, immobilized enzymes are preferred to overcome the limited properties of free enzymes, such as stability and reusability (Gennari et al., 2021). Enzyme immobilization methods involve covalent binding to a specific carrier, cross-linking, and encapsulation. However, the chemicals involved in these techniques could denature the enzyme and lead to the loss of its activity. In addition, the process of enzyme production and purification is time-consuming, laborious, and costly. Recently, researchers developed a cell surface display system, which uses synthetic biology techniques to design regenerable whole-cell biocatalysts that convey target enzymes on the surface of alive microbial cells (Dwevedi and Kayastha, 2012). The basic principle of the cell surface display technique involves tethering the target protein to an anchor protein (typically an outer membrane protein (Gilbert et al., 2014), which is integrated into the host cells and then actively transferred to the cell surface through the host’s cellular secretory system. There have been abundant published studies on the surface display of lipase and cutinase (Lee et al., 2005a,b; Liu et al., 2010; Chen et al., 2021). PETase also had been displayed successfully using yeast as chassis cells (Chen et al., 2020). The structure predicted by Gaetano et al. (Cristalli et al., 2000) suggests that the outer membrane-bound fatty acid transporter (FadL) of *E. coli* is expected to be rich in β structures and it can cross the outer membrane multiple times to form fatty acid-specific channels. FadL has previously been successfully used as an anchor peptide for esterase display on the surface of *E. coli* (Lee et al., 2004).

Hydrophobins are small, cysteine-rich proteins produced by filamentous fungi. They can spontaneously self-assemble into amphipathic monolayers at hydrophobic or hydrophilic interfaces resulting in a reversal of the surface wettability (Linder et al., 2005; Ren et al., 2013). Doris (Ribitsch et al., 2013) enhanced the PET-hydrolyzing activity of a bacterial cutinase by fusion to fungal hydrophobins to improve PET-hydrolyzing activity. The hydrolytic activity of PETase at 30°C was increased by 51% by fusing with hydrophobin RolA extracted from *Aspergillus oryzae* (Puspitasari et al., 2021). Kontkanen (Kontkanen et al., 2009) demonstrated a higher activity of a polyester hydrolase from *Coprinopsis cinerea* to cleave cutin and suberin in the presence of class II type of hydrophobin protein derived from *Trichoderma reesei* (HFBII) (Nakari-Setälä et al., 1997; Hakanpää et al., 2006; Dai et al., 2021). These findings caused us to presume that HFBII can also stimulate the enzymatic modification of synthetic polymers like PET. In this study, we constructed a hydrophobic cell surface display system, herein called HCSD, on the surface of *E. coli* by fusing PETase with hydrophobin HFBII and FadL. The expression system allows *E. coli* to be used as an efficient and sustainable whole-cell catalyst for the industrial degradation of PET.

## Materials and methods

### Bacteria and reagents

*Escherichia coli* BL21 (DE3) was incubated in LB medium supplemented with kanamycin (50 mg/ml) at 37°C in the shaker at 200 rpm unless otherwise specified. DNA polymerase, restriction enzymes, and the reagents used in cloning work were obtained from New England Biolabs (Ipswich, MA). The discarded PET drinking water bottles were cleaned by washing with double-distilled water and sterilized with ethanol (75%, v/v) before being used. *Escherichia coli* TOP10 was used in recombinant DNA cloning and plasmid propagation. *Escherichia coli* TOP10 and *Escherichia coli* BL21(DE3) competent cells were provided by TransGen Biotech Co., LTD (China). Protein experiment-related supplies and reagents were purchased from Thermo Fisher Scientific (Guangzhou, China). The plasmid Extraction Kit was purchased from Takara Bio Inc., (Shiga, Japan). Other chemicals used in this work were purchased from Sigma-Aldrich Trading Co. Ltd. (Shanghai, China).

### Construction of HCSD system

The vector used to construct the HCSD system (pET-30a (+): *HCSD*) was derived from PETase-containing plasmids constructed from PETase-only-containing plasmid pET-30a (+): *PETase* which was constructed in the previous study (Jia et al., 2021). The flexible linker (GGGGS)_3_, which can increase folding and stability and improve intrinsic biological activity (Chen et al., 2013), was used to connect the different domains. The gene encoding the PETase was cloned from this vector using the primer PETase-F (which contains (GGGGS)_3_-linker) and PETase-R. The fusion site on *fadL* was selected consistent with the previous reference to ensure the stability of PETase on the surface of *E. coli* (Lee et al., 2004). The FadL fragment was amplified by PCR from *E. coli* BL21 (DE3) using the primer FadL-F and FadL-R (which contains (GGGGS)_3_-linker). Based on the published coding DNA sequence of *hfbII* from *T. reesei,* the *hfbII* was fully synthesized after codon optimization conducted by GenScript Inc. (China). We additionally added a hexa-histidine tag C-terminally before the STOP codon using the primers HFBII-F and HFBII (Supplementary Table S1). The truncated *fadL* gene was cloned and linked to the *PETase* gene at the C-terminus, and the other end of the *PETase* was linked to the gene of hydrophobic protein. The Loop-opening vector fragment was generated using the primer P-F and its reverse complement P-R. Finally, the resulting large gene fragment was cloned into the plasmid fragment for expression using the Seamless Cloning and Assembly kit provided by TransGen Biotech Co., LTD (China) (the final sequences can be found in the Supplementary material). Based on the nucleotide sequence of HCSD, the molecular weight size of HCSD protein was calculated by the protein molecular weight prediction tool ProtParam tool to be about 81 kDa. The sequence of the resulting plasmids was confirmed using Sanger sequencing by SinoGenoMax Co., LTD (China). *E. coli* cells displaying PETase (CSD) or HFBII (HCSD-HFBII) alone were used as control groups. For HCSD-HFBII, the primers involved in connecting FadL and HFBII are HCSD-HFBIIF and HCSD-HFBIIR. The construction process of CSD is consistent with HCSD except that HFBII was not connected. Primers used in this work are listed in Supplementary Table S1.

### Cultivation and induction conditions

After that, the expression plasmids, pET-30a (+): *HCSD*, pET-30a (+): *CSD* and pET-30a (+): *HCSD-HFBII* were transformed into *E. coli* BL21(DE3) competent cells *via* heat shock. The successful transformation was confirmed *via* agarose gel electrophoresis and Sanger sequencing. 200 μl pre-cultures were inoculated from glycerol stocks and grown in a 10 ml LB medium overnight. After that, 1 ml pre-cultures were transferred to a 50 ml LB medium for expression. Cell growth of the cultures was measured using a HITACHI U-2910 spectrophotometer (Japan). After the cultures reached an OD_600_ of 3, induction was conducted at 18°C and 150 rpm using IPTG at final concentration of 0.6 mM. For the separation of free PETase, 50 ml culture medium was centrifuged for 5 min at 4°C. The collected *E. coli* cells were resuspended in 5 ml sodium phosphate (10 mM, pH 7.4). After that, the resuspended cells were disrupted by an ultrasonic homogenizer at 4°C. And finally, the crude enzyme solution containing PETase was collected *via* centrifugation at 4°C for 30 min and applied to the degradation process of PET directly.

### Characterization of the structure of HCSD system

#### Western blot

Outer membrane proteins were prepared according to the instructions of kits purchased from BestBio (Shanghai, China) (Item No. BB-3151). 10 μl whole-cell lysates or membrane fraction samples were loaded onto a 12% (wt/vol) SDS-PAGE. Western blot analysis was carried out following the standard procedure of Sambrook and Russell (2006). Anti-his mouse monoclonal antibodies [TransGen Biotech Co., LTD (China)] at a dilution rate of 1:1000 and rabbit anti-rat IgG (H + L) HRP conjugated secondary antibodies at a dilution rate of 1:500 was used to conduct the immunodetection of the hexa-histidine in the HCSD system and free PETase. A lumino light-emitting ECL kit (Thermo Scientific, United States) was used for band detection.

#### Immunofluorescence microscopy and cell viability assays

For immunofluorescence microscopy assays, 2 ml of *E. coli* cells were harvested by centrifugation at 4000 rpm (Centrifuge, 5,452, Eppendorf, Germany) for 5 min at 4°C after induction at 18°C for 24 h. The cell pellet was washed three times with phosphate-buffered saline (PBS) buffer (10 mM, pH 7.4) and resuspended in 300 μl PBS buffer supplemented with 1% (wt/vol) of bovine serum albumin (BSA) solution. Cells were incubated in 1 ml of anti-his mouse monoclonal antibody at a dilution rate of 1:300. After 4 h incubation at 4°C, cells were washed five times with PBS solution. The cell-antibody complex was then incubated overnight at 4°C with 1 ml of second antibody, Alexa Fluor 488-conjugated goat anti-mouse IgG (TransGen Biotech), at a dilution rate of 1:300. After washing with PBS buffer thoroughly, the image of cells was observed with the super-resolution confocal microscope Leica SP8 STED 3X. Cell viability within the biofilm was examined using a Live/Dead BacLight kit (Hympanova et al., 2020).

### Enzyme activity assay

#### Tributyrin plate clearing assay

Tributyrin plate clearing assays were performed based on previously described studies (Filloux and Ramos, 2014). Briefly, 30 ml of sterilized tributyrin emulsion, containing 50% (v/v) tributyrin and 50 g/l gum arabic in distilled water, was added to 0.97 l of melted LB solid medium which contained 50 g/l kanamycin and 0.6 mM IPTG. The mixture was mixed thoroughly, cooled to 60°C, and then poured into Petri plates. The plates were left to solidify for 20 min under UV in the laminar flow cabinet, then inoculated with 1 μl of *E. coli* cells containing pET-30a (+): *HCSD*, pET-30a (+): *CSD* and pET-30a (+): *HCSD-HFBII*, respectively, and incubated at 18°C for 72 h. It should be noted that the preparation of tributyrin emulsion requires thorough mixing with a homogenizer (Eppendorf, Germany).

#### Degradation of 4-nitrophenyl butyrate ester with HCSD system

The *E. coli* cells containing plasmids pET-30a (+): *HCSD*, pET-30a (+): *CSD* and pET-30a (+): *HCSD-HFBII* (OD_600_ = 3, 200 μl) were incubated with 3 ml of 4 mM 4-nitrophenyl butyrate ester (4-NPB) in 100 mM phosphate buffer (pH 7.5) for 2 min at 35°C. 4-NPB has been widely used as a model substrate to measure the activity of PETase (Qiao et al., 2022). The formation of p-nitrophenol (molar extinction coefficient = 16,600 M^−1^ cm^−1^ (Ji et al., 2021)) was monitored at a wavelength of 400 nm *via* a spectrophotometer (HITACHI U-2910, Japan).

#### Reusability and stability assays of the HCSD whole-cell biocatalyst

After 48 h of induction using IPTG, 10 ml *E. coli* cells containing plasmids pET-30a (+): *HCSD* and pET-30a (+): *CSD* were used to study the thermostability of HCSD and CSD systems, and unpurified crude PETase from 10 ml of cell culture was used as the control. After 7 days of incubation with 4 mM 4-NPB in 50 mM glycine-NaOH buffer (pH 7.0) using a static incubator at 40°C, the residual activity was measured every 24 h. The biocatalyst recycling was performed by collecting the *E. coli* cells which harbored plasmid pET-30a (+): *HCSD* and pET-30a (+): *CSD* using centrifugation at 3500 × g for 5 min. The activity was measured after washing the cells three times with 50 mM glycine-NaOH (pH 7.0). The initial catalytic activity was defined as 100%. The experiments were conducted in triplicates and values represent the mean ± standard error.

#### Degradation of pet film and fed-batch culture

PET film from a conventional bottle (NONGFU SPRING, 2 l, specific polymer characteristics unspecified) was cut into small pieces of 0.015 g about 1 cm^2^ square in area and used in subsequent fed-batch experiment. After completing the induction for 48 h in 2.3, the cell culture containing plasmids pET-30a (+): *HCSD*, pET-30a (+): *CSD* and pET-30a (+): *HCSD-HFBII* were incubated, respectively, with PET film in 30 ml of LB medium containing kanamycin (50 mg/ml), and 30 ml buffer containing of 50 mM Na_2_HPO_4_-HCl (pH 8.0) which was used for the degradation process of PETase. The method for PET degradation of PETase was consistent with the previous literature (Jia et al., 2021). The amount of free PETase produced by disrupting the *E. coli* cells with the same OD as the HCSD expression system at the induction time was used as a control. 30 ml LB medium was supplied to the degradation system of HCSD, and unpurified crude PETase from 30 ml of cell culture was added to the degradation system of PETase on the fourth day of the degradation process. The TPA content in the product was sampled every 12 h during the degradation process.

#### Degradation product (TPA) measurement

As described before, degradation products were detected by measuring absorbance at 260 nm using a G1315D diode array detector with the aid of a C18 column mounted on an Agilent 1200 LC system (Jia et al., 2021). Briefly, a mixture of 0.1% (v/v) formic acid (A) and acetonitrile (B) was used as the mobile phase at a flow rate of 0.8 ml/min to separate the analytes of interest. The mobile phase B was changed gradually from 1 to 5% for 5 min, 5–44% for 7 min, and 44–70% for 3 min, with a constant ratio of 95% for 10 min. The concentration of TPA, MHET and BHET was detected at a wavelength of 260 nm and quantified from the areas of the absorption peaks by standard curves.

#### SEM analysis

At the end of the PET film biodegradation period (7 days), the reactions were stopped by adding 5 ml of 3 M HCl. The PET pieces in the flasks were used for scanning electron microscopy (SEM) analysis by JSM-7800 (JEOL, Japan) after washing with 1% SDS, ultrapure water, and ethanol, respectively. In SEM analysis, samples were coated with Au for 180 s at 20 mA and were scanned under a low vacuum at 10 kV.

### Contact angle measurement

Degraded PET samples were separated from the cell cultures containing plasmids pET-30a (+): *HCSD*, pET-30a (+): *CSD*, pET-30a (+): *HCSD-HFBII* and empty plasmid pET-30a (+), incubated in 1% (w/v) SDS solution for 2 h, washed with deionized water, followed by sonication in 70% ethanol solution for 30 min, washed again with deionized water to remove any biofilm, and attached organics thoroughly, and finally dried naturally at room temperature. For contact angle measurements using Kruss DSA K100 (Kruss GmbH, Hamburg, Germany), 20 μl ultrapure water was added to the surface of PET, and the drop-shape images were recorded and analyzed using Drop Shape Analysis software (Kruss). For surface tension analysis of bacterial solution, 20 μl cell culture harboring pET-30a (+): *HCSD*, pET-30a (+): *CSD* and pET-30a (+): *HCSD-HFBII* with OD_600_ of 3 in LB medium was added to non-pretreated PET surface, and *E. coli* culture without any expression plasmids was used as a negative control. Subsequently, the contact angle between the cell cultures and PET is detected as before. Three positions of each film were analyzed to increase the reliability of experimental results. The results were expressed as mean ± standard deviation.

## Results and discussion

### Construction of HCSD expression system

The HCSD expression system was constructed by performing multiple gene fusions on the expression plasmid, pET 30a (+). Supplementary Schematic 1 illustrates the sandwich structure of the degradation part of HCSD. The expression vectors for HCSD and CSD contained the robust T7 promoter and its downstream lac manipulator sequence, and required the use of IPTG for induction. The plasmid map of the HCSD expression system is shown in Supplementary Figure 1.

### Characterization of the structure of the HCSD system

**Figure 1:**
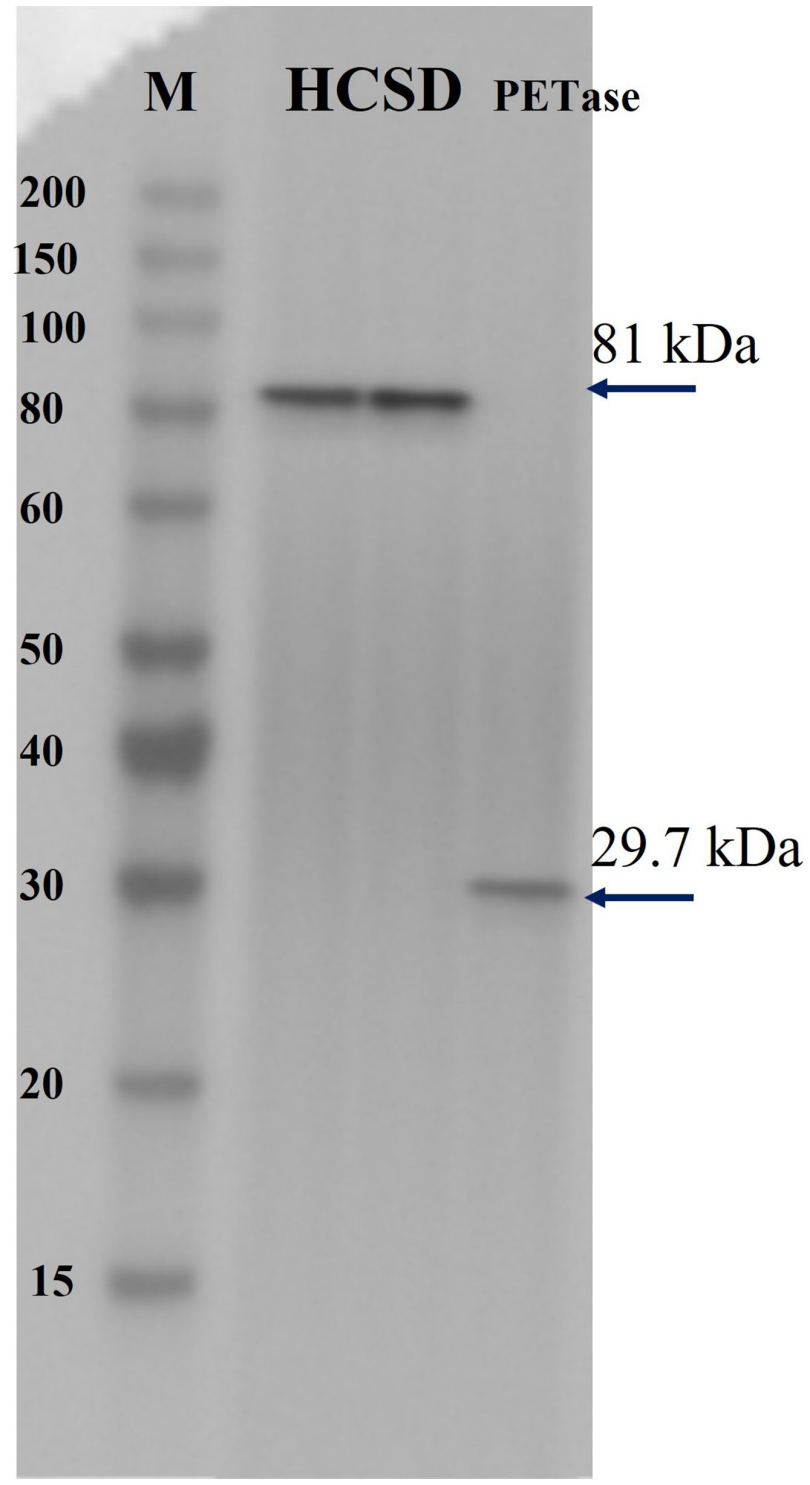
Immunoblotting of two HCSD proteins and PETase. Lane HCSD, Membrane protein extracts of *E. coli* BL21 (DE3) harboring pET-30a (+): *HCSD*; lane PETase, whole-cell lysates of *E. coli* BL21 (DE3) harboring pET-30a (+): *PETase.*

We demonstrated the successful construction of the HCSD system by Sanger sequencing. The membrane protein was extracted and confirmed by SDS-PAGE and western-blot analysis. Free PETase showed a molecular weight of only 30 kDa, and the fused protein in the HCSD system showed an approximate molecular weight of 81 kDa, which is consistent with the predicted result. The results indicated that the fusion protein is correctly expressed on the outer membrane of *E. coli,* as shown in Figure 1 and Supplementary Figure 2. In addition, as the expression location of the fused protein in the HCSD system is located on the outer membrane of *E. coli*, the protein expression level is so low that it needs to be induced at a higher cell concentration (OD_600_ = 3) and extracted from the membrane protein before it can be detected using SDS-PAGE and Western blot (Supplementary Figure 2).

**Figure 2:**
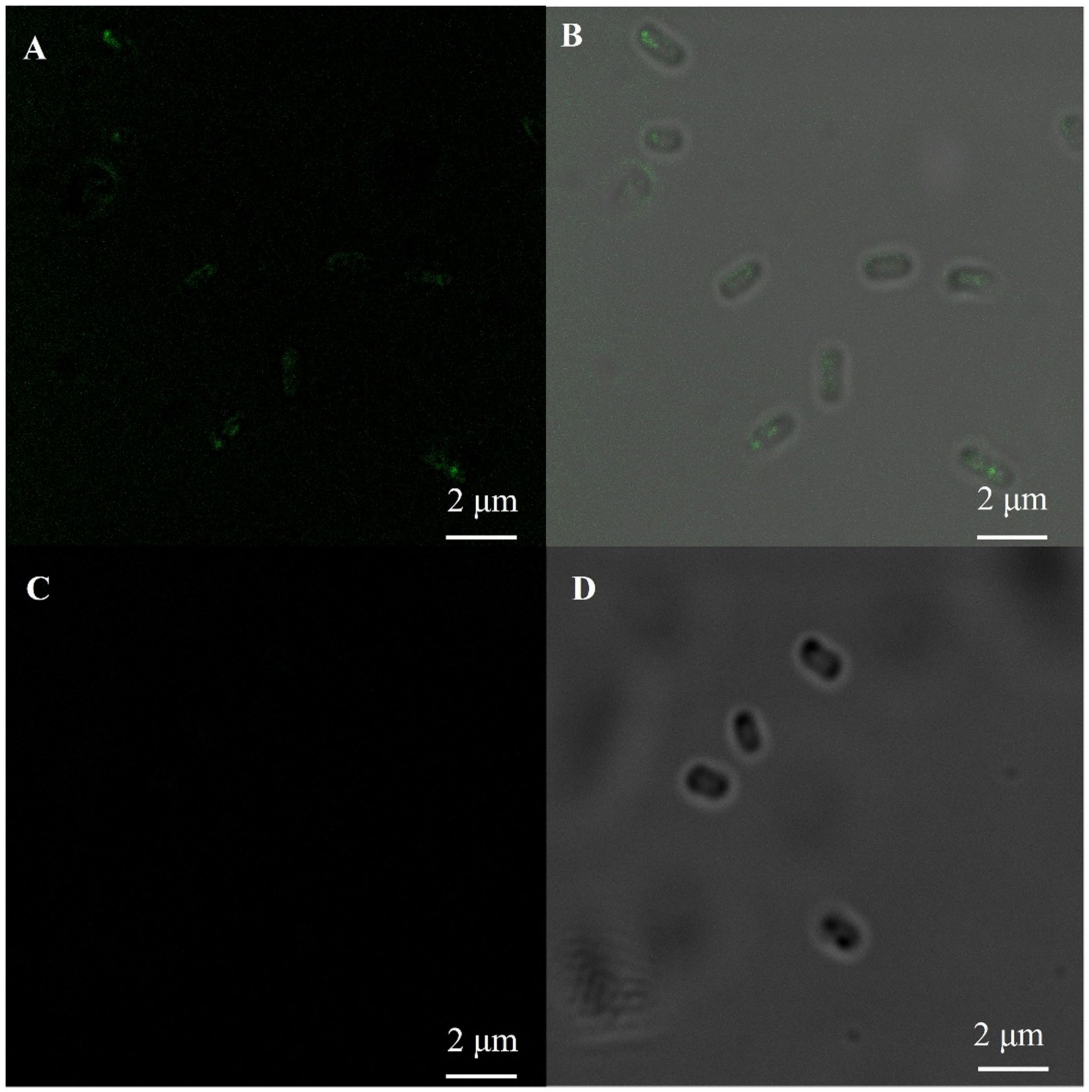
Immunofluorescent labeling of his-tag on the cell surface of *E. coli*. Fluorescence microscopy **(A,C)** and bright-field **(B,D)** images of PETase **(C,D)** and HCSD **(A,B)** displayed on *E. coli* cells. The *E. coli* cells were immunologically labeled with the anti-his mouse monoclonal antibody as the first antibody and rabbit IgG (H + L) HRP-conjugated anti-rat as the second anti-body.

The fluorescence immunoassay was performed by selecting a monoclonal antibody against the his-tag as the first antibody. The fluorescence was only detected on the surface of *E. coli* containing the HCSD expression system, while no fluorescence was observed in *E. coli* (DE3) which displays PETase only, as shown in Figure 2. This image together with Supplementary Figure 2 confirms the expression of HCSD on the outer cell membrane of *E. coli*.

### Characterization of enzyme activity and hydrophobicity of HCSD system

**Figure 3:**
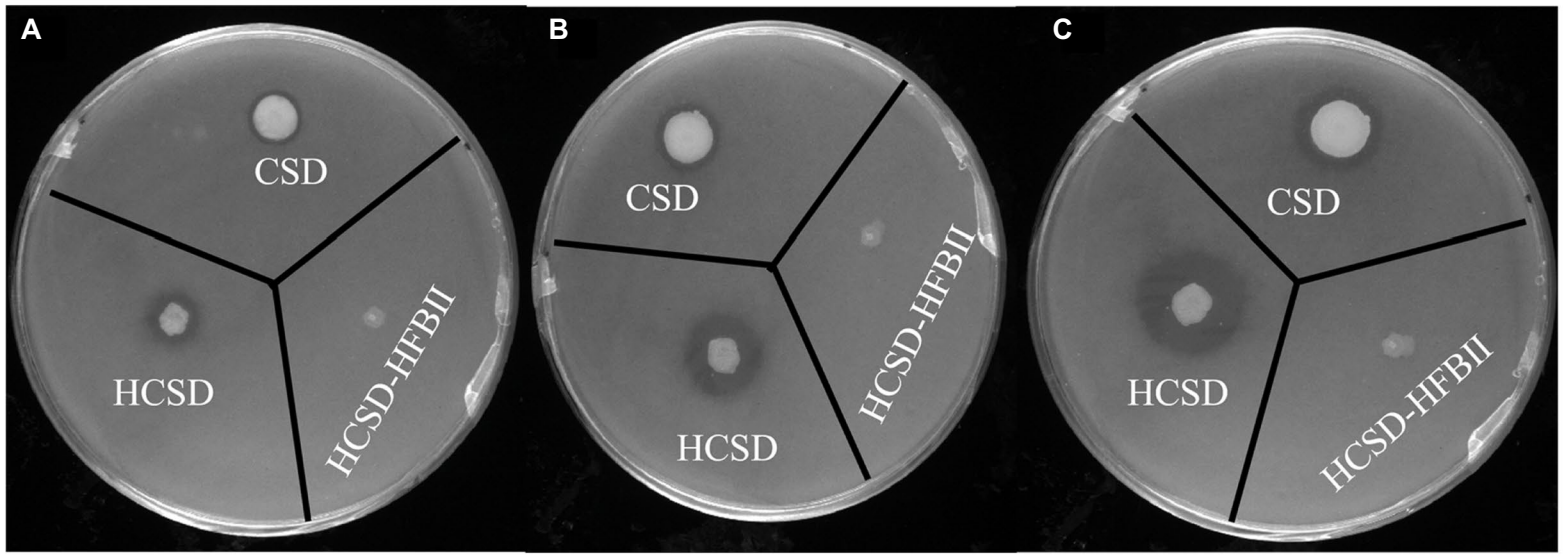
Halo formation of PETase-displaying *E. coli* BL21(DE3) harboring plasmid pET30a (+): *HCSD*, pET30a (+): *CSD* and pET30a (+): *HCSD-HFBII* on tributyrin agar plate incubated at 18°C after **(A)** 1 day; **(B)** 3 days; **(C)** 5 days.

Halo assay was conducted to test the enzymatic activity of HCSD. Figure 3 indicates that expanding clear halos were formed in the plates with tributyrin as substrate in HCSD and CSD groups, and they became more significant with time. In contrast, the *E. coli* colony in the control group, which contains the HCSD-HFBII expression system, neither grew well nor formed a clear halo since HFBII has no PETase activity. In addition, from the size of the clear halos, the HCSD expression system was higher in the enzyme activity with tributyrin as substrate, which indicated the presence of hydrophobic proteins contributes to the enzymatic activity. This is consistent with the predicted results and can be explained by the fact that the presence of hydrophobic proteins facilitates the binding of the enzyme and the substrate, thus promoting the hydrolysis of the substrate (Puspitasari et al., 2021).

**Figure 4:**
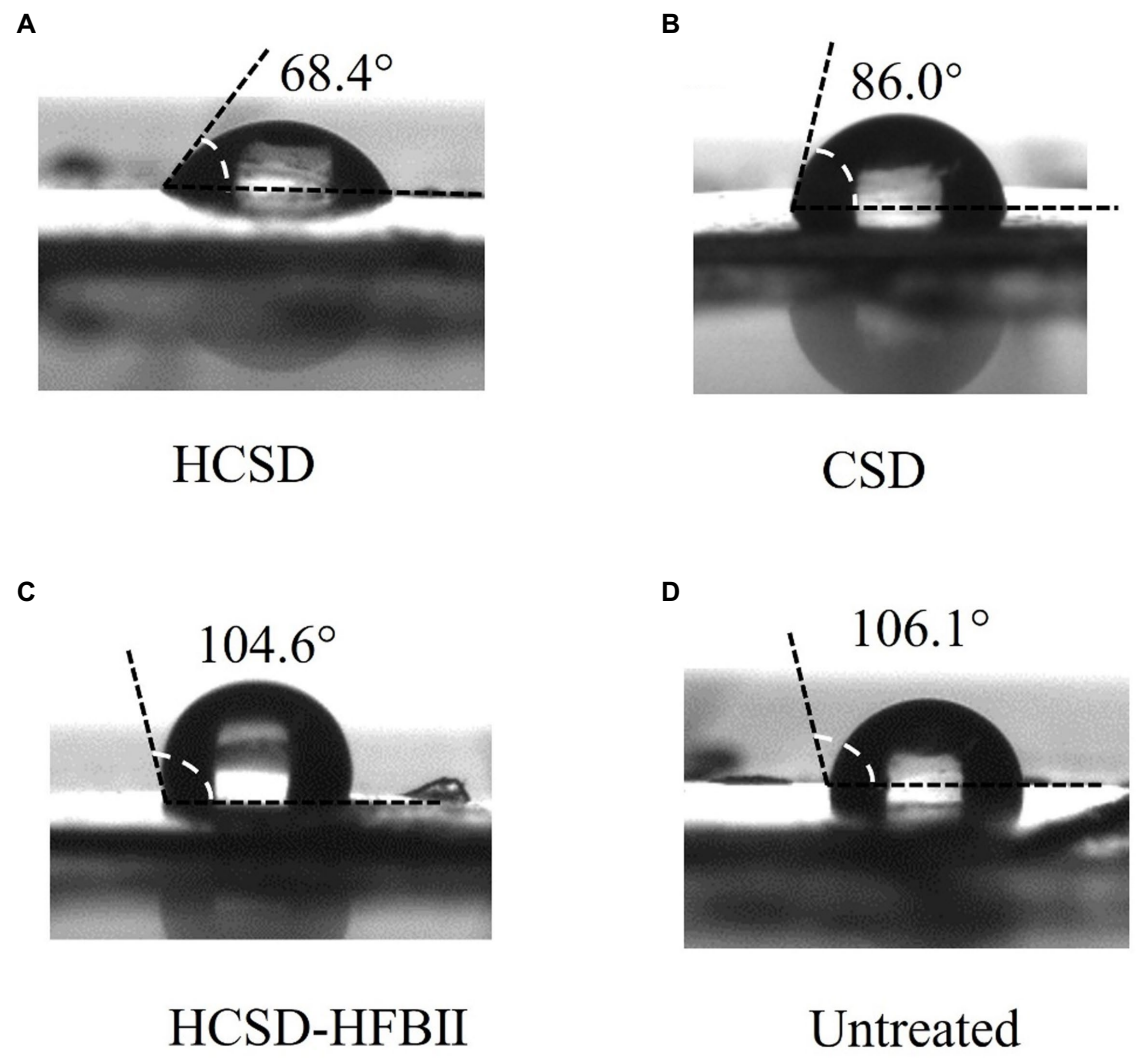
Water contact angle (WCA) of pretreated PET surface by *E. coli* harboring plasmid pET30a (+): HCSD **(A)**, pET30a (+): *CSD* **(B)** and pET30a (+): *HCSD-HFBII* **(C)** and untreated **(D)**.

It is well known that the degradation of PETase on PET changes the hydrophobic properties of the PET surface by exposing the hydrophilic groups (-OH and-COOH) due to the breakage of ester bonds in the molecular chains of PET (Giovambattista et al., 2007), and the changes can be monitored by measuring the contact angle with water. Contact angle measurements were conducted on *E. coli* digested PET samples, as shown in Figure 4. The water contact angle of non-pretreated PET was 106.1 ± 2°, while the contact angle was 68.4 ± 1° after treatment with *E. coli* containing the HCSD system. This indicated that the HCSD system significantly altered the hydrophobicity of PET due to the enzymatic digestion of PETase, which was exposed to the surface of *E. coli*. The PETase in CSD showed some capability of degradation (86.0 ± 1°), but less than HCSD system, which due to the self-assembly of HFBII would form an amphipathic layer on the PET surface, creating a hydrophilic surface that targets the PETase enzyme to the PET film. Moreover, PET treated with HCSD-HFBII showed a comparable contact angle (104.6 ± 1°) to the control group, indicating that HFBII itself did not have any degradation effect on PET, but it only enhanced the hydrolysis of PET by PETase.

**Figure 5:**
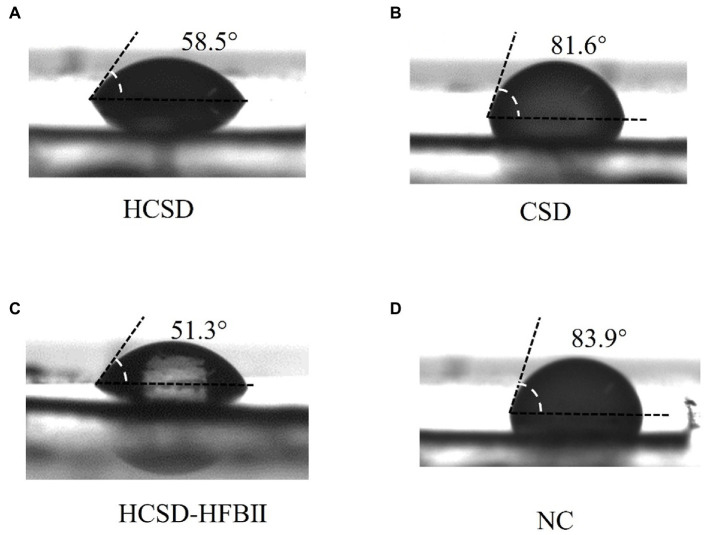
Surface tension analysis of *E. coli* harboring HCSD **(A)**, CSD **(B)** and HCSD-HFBII **(C)** with OD_600_ of 3 in LB medium of non-pretreated PET surface, *E. coli* without any expression plasmids **(D)** as a negative control.

The difference in contact angle with PET was measured between the HCSD, CSD and HCSD-HFBII expression systems for the same concentration of the *E. coli* solution. Figure 5 shows that the contact angle with PET was smaller for the bacterial solution containing the HCSD and HCSD-HFBII systems (58.5 ± 1° vs. 51.3 ± 2°), which could be attributed to the difference in the surface tension of the droplets. This difference in surface tension is due to the diverse hydrophilic and hydrophobic properties of the bacterial surface caused by the self-assembly of the HFBII (Wösten and de Vocht, 2000). Additionally, the bacterial solution containing the CSD system has a relative surface tension to the negative control group (81.6 ± 3° vs. 83.9 ± 2°), suggesting that the difference in surface tension is caused exclusively by the hydrophobic protein HFBII.

**Figure 6:**
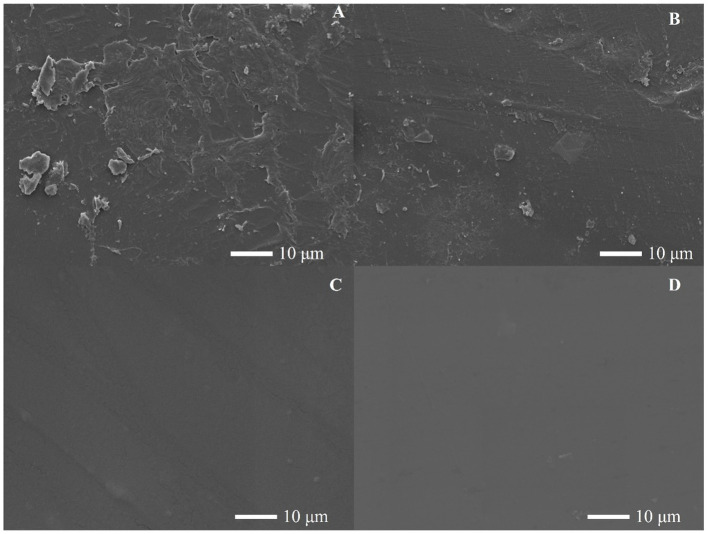
SEM images of the PET films surface treated by *E. coli* contained **(A)** HCSD; **(B)** CSD-PETase; **(C)** HCSD-HFBII expression system for 96 h, **(D)** PET film without any treatment.

The *E. coli* containing the HCSD, CSD and HCSD-HFBII expression system were co-incubated with PET film, and electron microscopic analysis showed that the PET film incubated with HCSD showed more significant grooves and holes compared to the *E. coli* (DE3) containing and CSD expression system (Figure 6). The cell surface display system displayed HFBII did not show any degradation effect. These results further illustrated that the hydrophobic protein HFBII can contribute to better degradation of PETase within the cell display system which could be illustrated that HFBII can colonize the PET surface and maintain high cell viability in biofilm (Supplementary Figure 3), thus resulting in better contact and act on PET (Huang et al., 2022).

**Figure 7:**
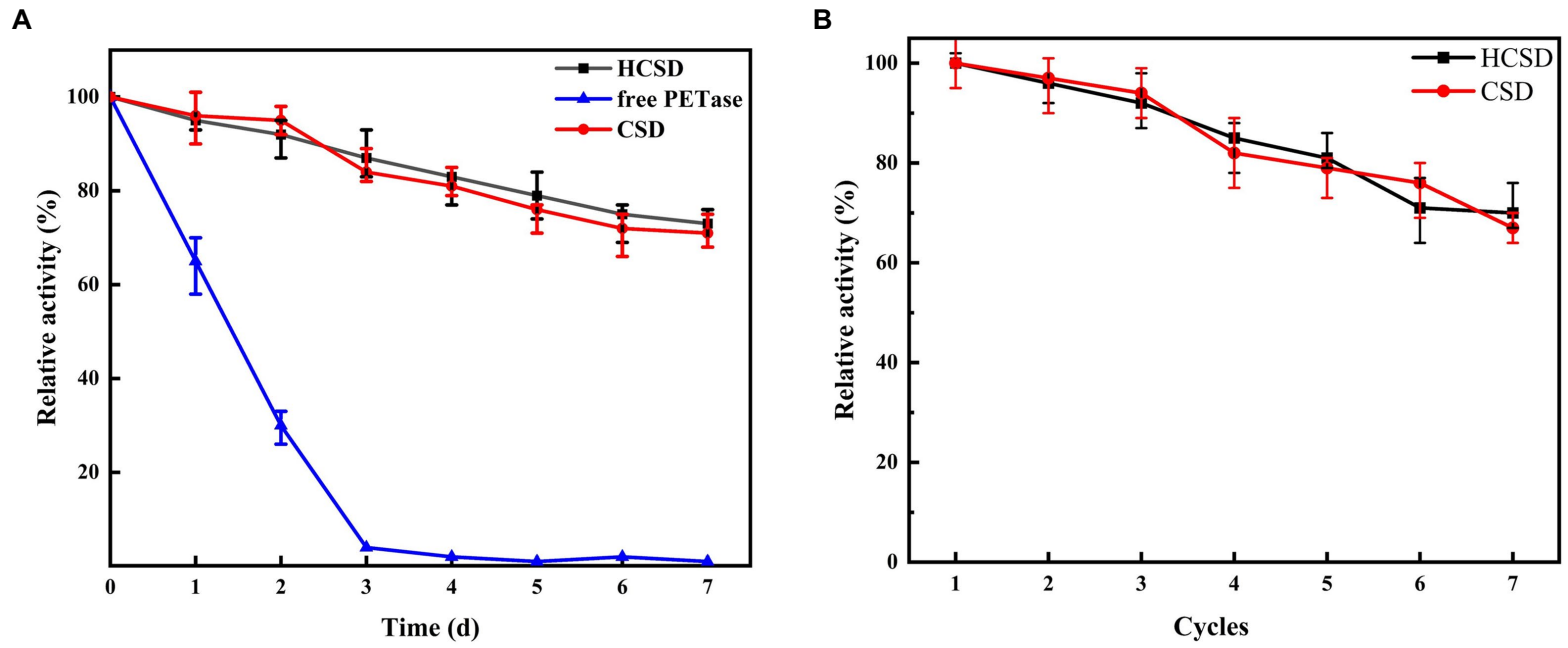
**(A)** The thermostability of PETase in HCSD and CSD systems compared to its free form. **(B)** Effect of recycling times on the hydrolytic activity PET f of PETase in HCSD and CSD systems. The enzyme activity was determined at 40°C in 50 mM glycine-NaOH buffer (pH 7.0) using as 4-NPB a substrate. Relative activity was calculated by assuming the initial activity as 100%.

The catalyst cost should be considered. The stability and reusability of the catalyst are crucial to reducing the catalyst cost before the constructed whole-cell catalysts are applied for industrial applications. Therefore, the sustainability of enzyme activity at 40°C and the recyclable times of the HCSD system were investigated. The thermostability of the HCSD whole-cell biocatalyst and free PETase at 40°C was evaluated as shown in Figure 7A. The activity of free PETase decreased rapidly with time, with only 65% of its initial activity and a complete loss of activity by the 3rd day, but the HCSD and CSD systems showed only a slight loss of activity, with residual activity remaining above 70% after the 7th day. Technically, the *E. coli* whole-cell catalyst recovery can be achieved simply by centrifugation. However, the uncertainty of the recovery process lies in whether the displayed PETase can continuously maintain its biological activity during this process. We measured the residual activity of the PETase in the HCSD and CSD systems during each cycle (Figure 7B). Overall, the result showed a low loss of PETase during the recycling process, which maintained 70% of its activity for seven cycling rounds, indicating that PETase has strong stability in the cell surface display systems. The reusability and sustainability of the whole-cell biocatalysts not only overcome the difficulty of recovering PETase from the reaction system without a complex and expensive purification process, but also make it suitable for the development of time-saving and profitable biocatalyst processes.

**Figure 8:**
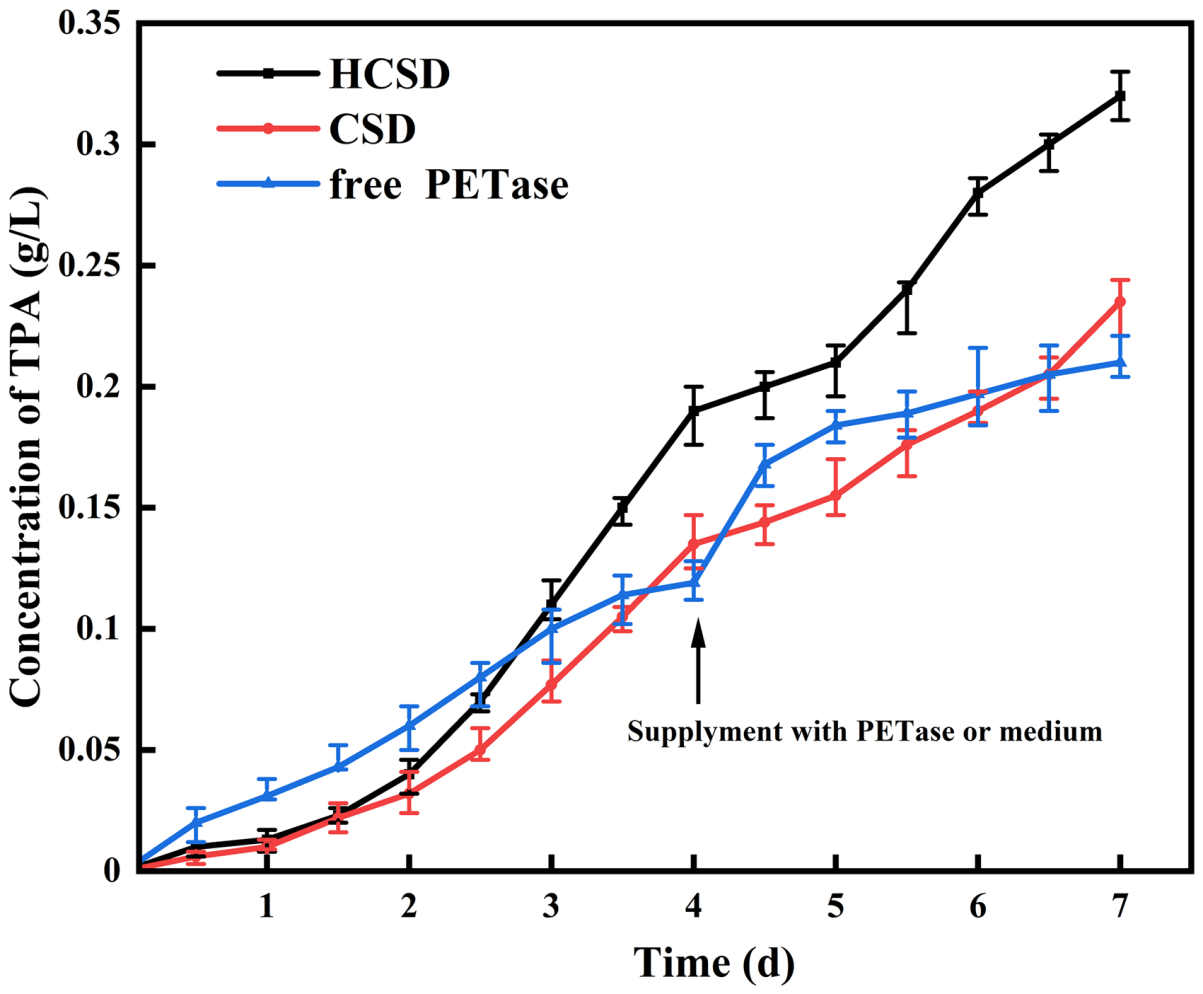
Accumulation of TPA in the reaction solution of PET film degradation at different degradation time. On the fourth day, 30 ml LB medium was supplied to the degradation system of HCSD and CSD, and unpurified crude PETase from 30 ml of cell culture was added to the degradation system of free PETase at the same time.

Samples were collected at different time intervals during the degradation process of 7 days, and the concentration of TPA was measured to confirm the effect of the HCSD system on PET degradation. As shown in Figure 8, when PET is degraded directly with free PETase, the reaction rate is faster than HCSD and CSD systems for some time in the beginning, and the amount of TPA released is three times higher than that of the HCSD degradation system on the 1st day. This is probably because the two expression systems involve the expression of membrane proteins, which are usually much lower than intracellular expression systems. Moreover, it costs some time to induce the expression of a sufficient amount of PETase in both HCSD and CSD systems. Surprisingly, when the degradation rate of the free PETase decreased with time, the activity of PETase within the HCSD system remained stable due to the enzyme was immobilized on the supporting cells. Furthermore, the higher hydrophilicity of the HCSD system resulted in a higher chance of microbial colonization and subsequent biodegradation. On the 4th day, the HCSD system was supplemented with an equal volume of LB medium, while the control group was supplemented with the same amount of PETase as at the beginning of the reaction. It can be seen that the degradation rate of the HCSD gradually increased due to bacterial growth after supplementation of the medium. In contrast, the degradation rate of the supplemented enzyme group was decreased until PETase was inactivated after only a short period of increase. In the long-term degradation process of 7 days, the HCSD expression system produced 52% more degradation products than the group where PET is degraded directly with free PETase due to the presence of hydrophobic proteins, which increase the affinity of PET and PETase.

## Conclusion

In summary, we have developed, for the first time, a whole-cell biocatalysis strategy based on the immobilization of PETase and coupling with hydrophobic protein on the surface of *E. coli* cells. We found that PETase could be displayed with functionality on the surface of *E. coli*. Furthermore, the whole-cell biocatalysts containing HCSD could be reused several times and achieve continuous degradation of PET without significant activity loss. Based on our research, more whole-cell biocatalysts containing HCSD expression systems can be developed and optimized in terms of enzyme type, linkers and anchor motifs selection in the future. This technology has a broad application prospect for the industrial degradation of PET waste plastics as it avoids the expensive enzyme separation and purification process.

## Data availability statement

The datasets presented in this study can be found in online repositories. The names of the repository/repositories and accession number(s) can be found in the article/Supplementary material.

## Author contributions

YJ: conceptualization, methodology, validation, data curation, writing original draft, and review and editing. NS, XH, ZC, and QW: review and editing. JX: conceptualization, review and editing, supervision, and project administration. All authors contributed to the article and approved the submitted version.

## Funding

The authors gratefully acknowledge the financial support provided by the National Natural Science Foundation of China (grant numbers 31961133017, 31961133018, and 31961133019). These grants are part of “MIXed plastics biodegradation and UPcycling using microbial communities” MIX-UP research project, which is a joint NSFC and EU H2020 collaboration. In Europe, MIX-UP has received funding from the European Union’s Horizon 2020 research and innovation programme under grant agreement No. 870294.

## Conflict of interest

The authors declare that the research was conducted in the absence of any commercial or financial relationships that could be construed as a potential conflict of interest.

## Publisher’s note

All claims expressed in this article are solely those of the authors and do not necessarily represent those of their affiliated organizations, or those of the publisher, the editors and the reviewers. Any product that may be evaluated in this article, or claim that may be made by its manufacturer, is not guaranteed or endorsed by the publisher.
