# Supplementary material for: Hydrophobic cell surface display system of PETase as a sustainable biocatalyst for PET degradation
Source: Front Microbiol. 2022 Sep 29;13:1005480. doi: 10.3389/fmicb.2022.1005480 (PMC9559558; doi:10.3389/fmicb.2022.1005480)
Supplement: Supplementary file 1 [file Data_Sheet_1.docx]

**Hydrophobic cell surface display system (HCSD) of *Is*PETase as a sustainable biocatalyst for PET degradation**

**Yunpu Jia^1,2^, Nadia A. Samak^3^, Xuemi Hao^1,2^, Zheng Chen^1,2^, Qifeng Wen^1,2^, Jianmin Xing^1,2,4*^**

^1^CAS Key Laboratory of Green Process and Engineering, State Key Laboratory of Biochemical Engineering, Institute of Process Engineering, Chinese Academy of Sciences, Beijing 100190, PR China

^2^College of Chemical Engineering, University of Chinese Academy of Sciences, Beijing 100049, PR China

^3^Environmental microbiology and biotechnology, Aquatic microbiology, University of Duisburg-Essen, 45141 Essen, Germany

^4^Chemistry and Chemical Engineering Guangdong Laboratory, Shantou 515031, PR China


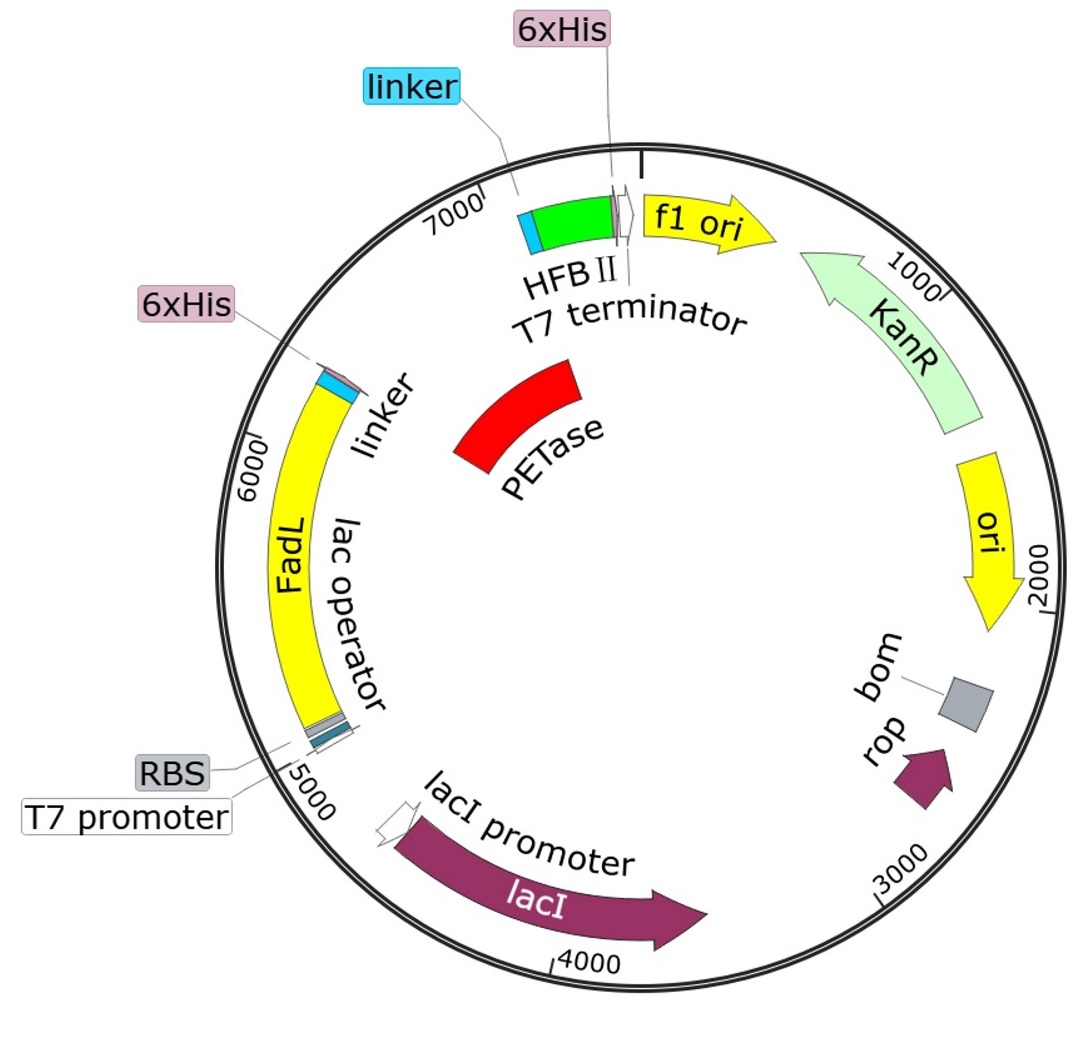
*** Correspondence:**Jianmin Xing
jmxing@ipe.ac.cn

**Supplementary Fig 1.** Maps of the constructed recombinant plasmids for the HCSD system.


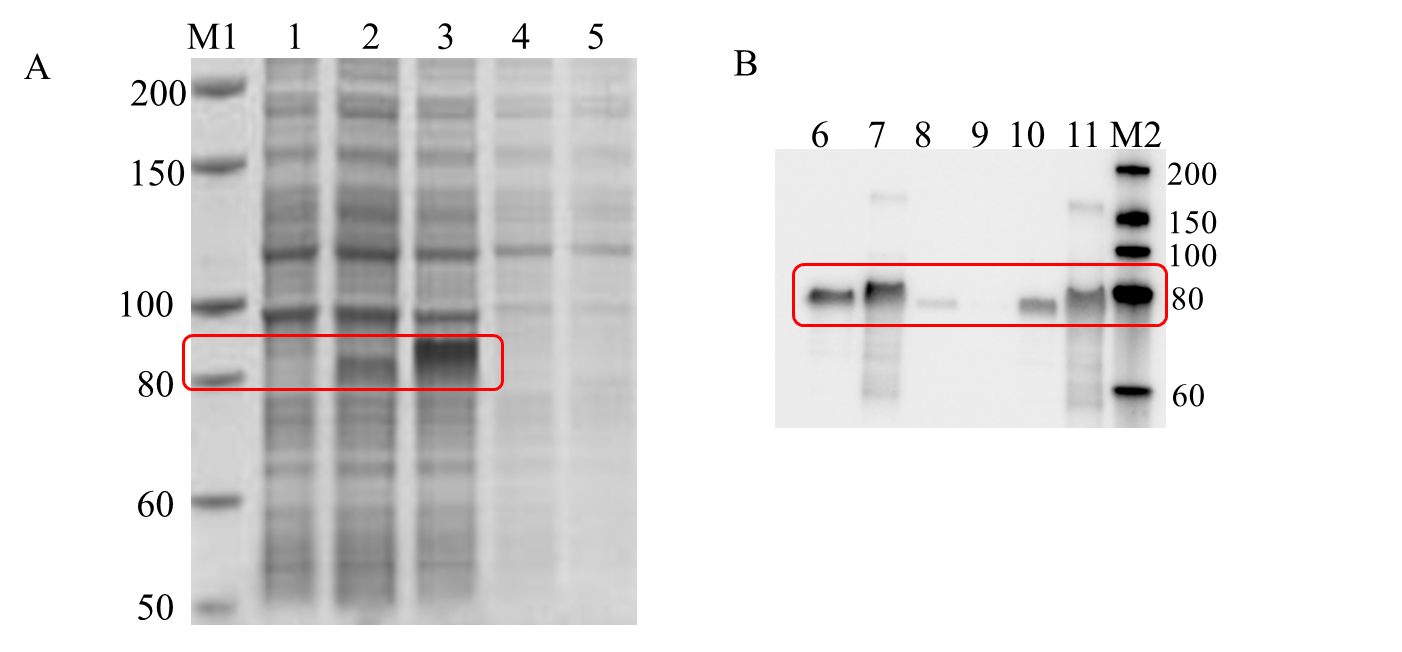


**Supplementary Fig 2.** SDS-PAGE (A) and western blot (B) analysis for HCSD cloned in pET-30a (+) and expressed in *E. coli* BL21(DE3) strain. M1: Protein marker, M2: Western blot marker, 1 and 8: Debris of cell lysate with induction for 24 h at OD_600_ = 0.5, 2 and 6: Debris of cell lysate with induction for 24 h at OD_600_ = 3, 3 and 7: Membrane proteins with induction for 24 h at OD_600_ = 3, 4: Supernatant of cell lysate with induction for 24 h at OD_600_ = 0.5, 5 and 9: Supernatant of cell lysate with induction for 24 h at OD_600_ = 3, 10: Membrane proteins with induction for 24 h at OD_600_ = 2, 11: Membrane proteins with induction for 24 h at OD_600_ = 4. The bands of target protein in HCSD system are in red solid line box. Debris of cell lysate and supernatant of cell lysate were obtained by one-step ultrasonic crushing while membrane proteins were extracted from whole cells using membrane protein kits.


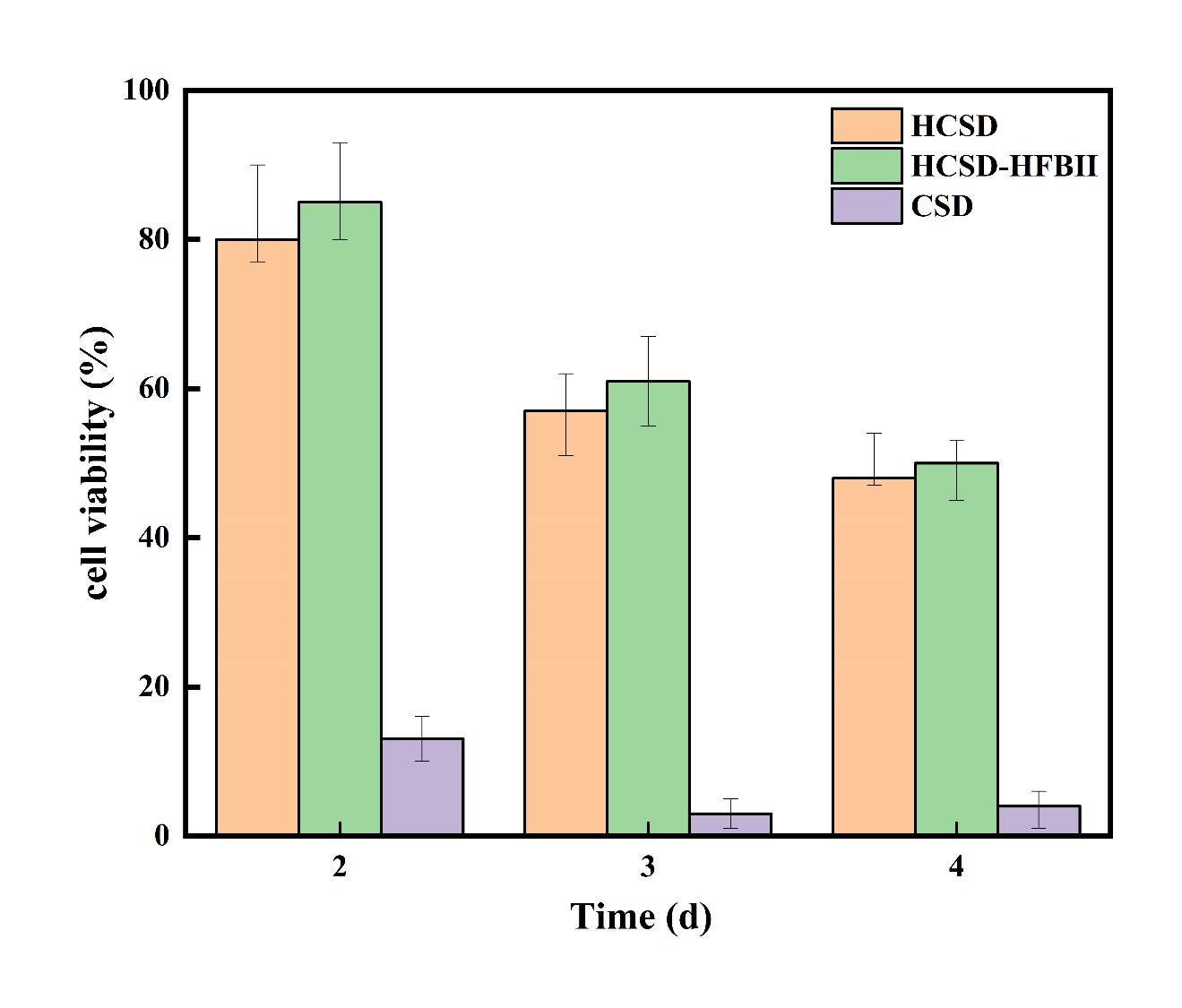


**Supplementary Fig 3. Proportion of live *E. coli* BL21(DE3) harboring plasmid pET30a (+): *HCSD*, pET30a (+): *CSD* and pET30a (+): *HCSD-HFBⅡ* on PET**

**Table S1. Primers used in this work.**

| Name | Sequence |
| --- | --- |
| FadL-F | TTAACTTTAAGAAGGAGATATACATATGAGCCAGAAAACCCTGTT |
| FadL-R | GTGATGATGATGATGGTGCATAGAGCCACCGCCACCACTTCCGCCTCCACCTGAACCTCCCCCCCCACGATTCTGTGCAGGAACTG |
| PETase-F | CAGTTCCTGCACAGAATCGTGGGGGGGGAGGTTCAGGTGGAGGCGGAAGTGGTGGCGGTGGCTCTATGCACCATCATCATCATCAC |
| PETase -R | CACCTGAACCTCCCCCCCCGCTACAGTTGGCGGTGCGGA |
| HFBⅡ-F | TCCGCACCGCCAACTGTAGCGGGGGGGGAGGTTCAGGTG |
| HFBⅡ-R | CCAAGGGGTTATGCTAGTTATTAGTGATGGTGGTGGTGATGA |
| P-F | TCATCACCACCACCATCACTAATAACTAGCATAACCCCTTGG |
| P-R | AACAGGGTTTTCTGGCTCATATGTATATCTCCTTCTTAAAGTTAA |
| HCSD-HFBIIF | CAGTTCCTGCACAGAATCGTGGGGGGGGAGGTTCAGGTG |
| HCSD-HFBIIR | CACCTGAACCTCCCCCCCC ACGATTCTGTGCAGGAACTG |

**Nucleotide Sequence Information**

**HFBII Nucleotide Sequence:**

GGGGGGGGAGGTTCAGGTGGAGGCGGAAGTGGTGGCGGTGGCTCTATGCAGTTTTTCGCGGTGGCCTTGTTCGCCACCAGCGCACTGGCAGCTGTCTGTCCGACTGGTCTGTTCAGCAATCCGCTGTGCTGCGCGACGAACGTGCTGGATCTGATTGGTGTTGACTGTAAAACCCCAACCATTGCTGTGGACACCGGCGCAATCTTTCAGGCGCATTGTGCGAGCAAGGGTTCCAAGCCGCTCTGCTGCGTTGCTCCGGTTGCGGATCAAGCGTTGTTATGCCAAAAAGCGATCGGCACCTTT**CATCACCACCACCATCAC**TAA

**FadL Nucleotide Sequence:**

ATGAGCCAGAAAACCCTGTTTACAAAGTCTGCTCTCGCAGTCGCAGTGGCACTTATCTCCACCCAGGCCTGGTCGGCAGGCTTTCAGTTAAACGAATTTTCTTCCTCTGGCCTGGGCCGGGCTTATTCAGGGGAAGGCGCAATTGCCGATGATGCAGGTAACGTCAGCCGTAACCCCGCATTGATTACTATGTTTGACCGCCCGACATTTTCTGCGGGTGCGGTTTATATTGACCCGGATGTAAATATCAGCGGAACGTCTCCATCTGGTCGTAGCCTGAAAGCCGATAACATCGCGCCTACGGCATGGGTTCCGAACATGCACTTTGTTGCACCGATTAACGACCAATTTGGTTGGGGCGCTTCTATTACCTCTAACTATGGTCTGGCTACAGAGTTTAACGATACTTATGCAGGCGGCTCTGTCGGGGGTACAACCGACCTTGAAACCATGAACCTGAACTTAAGCGGTGCGTATCGCTTAAATAATGCATGGAGCTTTGGTCTTGGTTTCAACGCCGTCTACGCTCGCGCGAAAATTGAACGTTTCGCAGGCGATCTGGGGCAGTTGGTTGCTGGCCAAATTATGCAATCTCCTGCTGGCCAAACTCAGCAAGGGCAAGCATTGGCAGCTACCGCCAACGGTATTGACAGTAATACCAAAATCGCTCATCTGAACGGTAACCAGTGGGGCTTTGGCTGGAACGCCGGAATCCTGTATGAACTGGATAAAAATAACCGCTATGCACTGACCTACCGTTCTGAAGTGAAAATTGACTTCAAAGGTAACTACAGCAGCGATCTTAATCGTGCGTTTAATAACTACGGTTTGCCAATTCCTACCGCGACAGGTGGCGCAACGCAATCGGGTTATCTGACGCTGAACCTGCCTGAAATGTGGGAAGTGTCAGGTTATAACCGTGTTGATCCACAGTGGGCGATTCACTATAGCCTGGCTTACACCAGCTGGAGTCAGTTCCAGCAGCTGAAAGCGACCTCAACCAGTGGCGACACGCTGTTCCAGAAACATGAAGGCTTTAAAGATGCTTACCGCATCGCGTTGGGTACCACTTATTACTACGATGATAACTGGACCTTCCGTACCGGTATCGCCTTTGATGACAGCCCAGTTCCTGCACAGAATCGTGGGGGGGGAGGTTCAGGTGGAGGCGGAAGTGGTGGCGGTGGCTCT

**PETase** **Nucleotide Sequence:**

ATG**CACCATCATCATCATCAC**CAAACGAACCCGTACGCACGCGGTCCGAATCCGACGGCAGCCTCTCTGGAAGCAAGCGCAGGTCCGTTTACGGTTCGTTCTTTCACCGTCTCTCGCCCGAGTGGCTATGGTGCAGGCACGGTGTATTACCCGACCAATGCTGGCGGTACGGTGGGTGCTATTGCGATCGTTCCGGGCTACACCGCGCGTCAGAGCTCTATTAAATGGTGGGGTCCGCGTCTGGCATCTCATGGTTTTGTGGTTATTACCATCGATACGAATAGTACCCTGGACCAGCCGAGTTCCCGTTCATCGCAGCAAATGGCGGCCCTGCGCCAAGTTGCAAGCCTGAACGGTACCAGCTCTAGTCCGATTTATGGCAAAGTCGATACGGCTCGTATGGGTGTGATGGGTTGGTCCATGGGCGGTGGCGGTTCCCTGATCTCAGCAGCTAACAATCCGAGCCTGAAAGCCGCGGCACCGCAGGCACCGTGGGATTCCTCAACCAATTTTTCGAGCGTCACGGTGCCGACCCTGATTTTCGCCTGCGAAAACGATTCAATCGCCCCGGTTAATTCTAGTGCACTGCCGATTTATGACTCCATGTCACGCAACGCGAAACAGTTTCTGGAAATCAATGGCGGTTCGCACAGCTGTGCTAACTCGGGTAACAGCAATCAAGCGCTGATCGGCAAGAAAGGCGTGGCCTGGATGAAACGTTTCATGGATAACGACACGCGCTACAGTACCTTTGCTTGTGAAAACCCGAACTCCACCCGTGTGTCTGACTTCCGCACCGCCAACTGTAGC

The linker sequence is underlined; His-tag peptide sequence is in bold.

**Full sequence of HCSD:**

ATGAGCCAGAAAACCCTGTTTACAAAGTCTGCTCTCGCAGTCGCAGTGGCACTTATCTCCACCCAGGCCTGGTCGGCAGGCTTTCAGTTAAACGAATTTTCTTCCTCTGGCCTGGGCCGGGCTTATTCAGGGGAAGGCGCAATTGCCGATGATGCAGGTAACGTCAGCCGTAACCCCGCATTGATTACTATGTTTGACCGCCCGACATTTTCTGCGGGTGCGGTTTATATTGACCCGGATGTAAATATCAGCGGAACGTCTCCATCTGGTCGTAGCCTGAAAGCCGATAACATCGCGCCTACGGCATGGGTTCCGAACATGCACTTTGTTGCACCGATTAACGACCAATTTGGTTGGGGCGCTTCTATTACCTCTAACTATGGTCTGGCTACAGAGTTTAACGATACTTATGCAGGCGGCTCTGTCGGGGGTACAACCGACCTTGAAACCATGAACCTGAACTTAAGCGGTGCGTATCGCTTAAATAATGCATGGAGCTTTGGTCTTGGTTTCAACGCCGTCTACGCTCGCGCGAAAATTGAACGTTTCGCAGGCGATCTGGGGCAGTTGGTTGCTGGCCAAATTATGCAATCTCCTGCTGGCCAAACTCAGCAAGGGCAAGCATTGGCAGCTACCGCCAACGGTATTGACAGTAATACCAAAATCGCTCATCTGAACGGTAACCAGTGGGGCTTTGGCTGGAACGCCGGAATCCTGTATGAACTGGATAAAAATAACCGCTATGCACTGACCTACCGTTCTGAAGTGAAAATTGACTTCAAAGGTAACTACAGCAGCGATCTTAATCGTGCGTTTAATAACTACGGTTTGCCAATTCCTACCGCGACAGGTGGCGCAACGCAATCGGGTTATCTGACGCTGAACCTGCCTGAAATGTGGGAAGTGTCAGGTTATAACCGTGTTGATCCACAGTGGGCGATTCACTATAGCCTGGCTTACACCAGCTGGAGTCAGTTCCAGCAGCTGAAAGCGACCTCAACCAGTGGCGACACGCTGTTCCAGAAACATGAAGGCTTTAAAGATGCTTACCGCATCGCGTTGGGTACCACTTATTACTACGATGATAACTGGACCTTCCGTACCGGTATCGCCTTTGATGACAGCCCAGTTCCTGCACAGAATCGTGGGGGGGGAGGTTCAGGTGGAGGCGGAAGTGGTGGCGGTGGCTCTATGCACCATCATCATCATCACCAAACGAACCCGTACGCACGCGGTCCGAATCCGACGGCAGCCTCTCTGGAAGCAAGCGCAGGTCCGTTTACGGTTCGTTCTTTCACCGTCTCTCGCCCGAGTGGCTATGGTGCAGGCACGGTGTATTACCCGACCAATGCTGGCGGTACGGTGGGTGCTATTGCGATCGTTCCGGGCTACACCGCGCGTCAGAGCTCTATTAAATGGTGGGGTCCGCGTCTGGCATCTCATGGTTTTGTGGTTATTACCATCGATACGAATAGTACCCTGGACCAGCCGAGTTCCCGTTCATCGCAGCAAATGGCGGCCCTGCGCCAAGTTGCAAGCCTGAACGGTACCAGCTCTAGTCCGATTTATGGCAAAGTCGATACGGCTCGTATGGGTGTGATGGGTTGGTCCATGGGCGGTGGCGGTTCCCTGATCTCAGCAGCTAACAATCCGAGCCTGAAAGCCGCGGCACCGCAGGCACCGTGGGATTCCTCAACCAATTTTTCGAGCGTCACGGTGCCGACCCTGATTTTCGCCTGCGAAAACGATTCAATCGCCCCGGTTAATTCTAGTGCACTGCCGATTTATGACTCCATGTCACGCAACGCGAAACAGTTTCTGGAAATCAATGGCGGTTCGCACAGCTGTGCTAACTCGGGTAACAGCAATCAAGCGCTGATCGGCAAGAAAGGCGTGGCCTGGATGAAACGTTTCATGGATAACGACACGCGCTACAGTACCTTTGCTTGTGAAAACCCGAACTCCACCCGTGTGTCTGACTTCCGCACCGCCAACTGTAGCGGGGGGGGAGGTTCAGGTGGAGGCGGAAGTGGTGGCGGTGGCTCTATGCAGTTTTTCGCGGTGGCCTTGTTCGCCACCAGCGCACTGGCAGCTGTCTGTCCGACTGGTCTGTTCAGCAATCCGCTGTGCTGCGCGACGAACGTGCTGGATCTGATTGGTGTTGACTGTAAAACCCCAACCATTGCTGTGGACACCGGCGCAATCTTTCAGGCGCATTGTGCGAGCAAGGGTTCCAAGCCGCTCTGCTGCGTTGCTCCGGTTGCGGATCAAGCGTTGTTATGCCAAAAAGCGATCGGCACCTTTCATCACCACCACCATCAC
