# Supplementary material for: Hydrophobic cell surface display system of PETase as a sustainable biocatalyst for PET degradation
Source: Front Microbiol. 2022 Sep 29;13:1005480. doi: 10.3389/fmicb.2022.1005480 (PMC9559558; doi:10.3389/fmicb.2022.1005480)
Supplement: Supplementary file 2 [file Image_1.pdf]

Hydrophobic cell surface display system (HCSD) of *Is*PETase as a sustainable biocatalyst for PET degradation

Yunpu Jia[1,2], Nadia A. Samak[3], Xuemi Hao[1,2], Zheng Chen[1,2], Qifeng Wen[1,2], Jianmin Xing[1,2,4*]

[1]CAS Key Laboratory of Green Process and Engineering, State Key Laboratory of Biochemical Engineering, Institute of Process Engineering, Chinese Academy of Sciences, Beijing 100190, PR China

[2]College of Chemical Engineering, University of Chinese Academy of Sciences, Beijing 100049, PR China

[3]Environmental microbiology and biotechnology, Aquatic microbiology, University of Duisburg-Essen, 45141 Essen, Germany

[4]Chemistry and Chemical Engineering Guangdong Laboratory, Shantou 515031, PR China

*** Correspondence:**
Jianmin Xing
jmxing@ipe.ac.cn

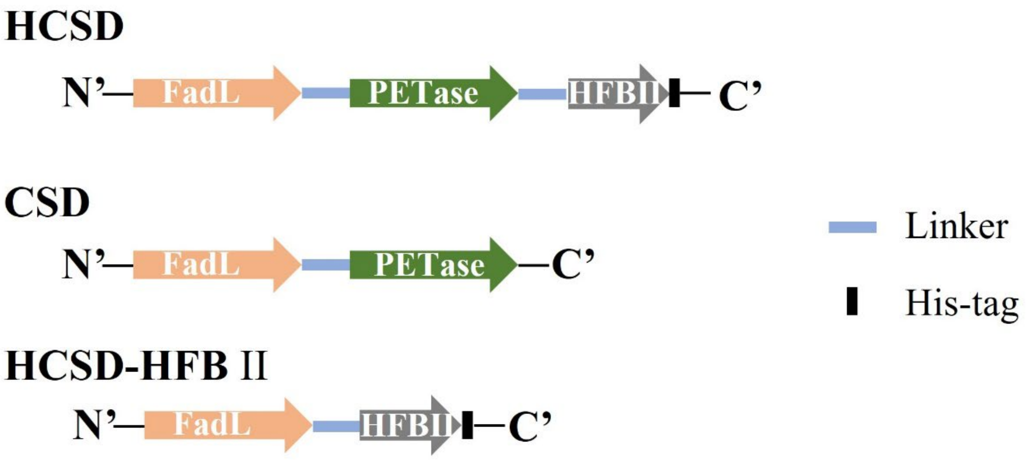

Schematic 1. Protein schemes for the HCSD, CSD and HCSD-HFBII constructs
